# Sex Differences in the Impact of the Mediterranean Diet on LDL Particle Size Distribution and Oxidation

**DOI:** 10.3390/nu7053705

**Published:** 2015-05-15

**Authors:** Alexandra Bédard, Louise Corneau, Benoît Lamarche, Sylvie Dodin, Simone Lemieux

**Affiliations:** 1 Institute of Nutrition and Functional Foods (INAF), 2440 Hochelaga Boulevard, Laval University, Québec, QC G1V 0A6, Canada; E-Mails: alexandra.bedard.1@ulaval.ca (A.B.); louise.corneau@fsaa.ulaval.ca (L.C.); benoit.lamarche@fsaa.ulaval.ca (B.L.); sylvie.dodin@fmed.ulaval.ca (S.D.); 2 School of Nutrition, Pavillon Paul-Comtois, 2425 rue de l’Agriculture, Laval University, Québec, QC G1V 0A6, Canada; 3 Department of Obstetrics and Gynaecology, Pavillon Ferdinand-Vandry, 1050 Medicine Avenue, Laval University, Québec, QC G1V 0A6, Canada

**Keywords:** Mediterranean diet, LDL size, oxidized LDL, men, women

## Abstract

Sex differences have been previously highlighted in the cardioprotective effects of the Mediterranean diet (MedDiet). The objective of this study was to investigate whether sex differences also exist with regard to LDL particle size distribution and oxidation. Participants were 37 men and 32 premenopausal women (24–53 years) with slightly elevated LDL-C concentrations (3.4–4.9 mmol/L) or total cholesterol/HDL-C ≥5.0. Variables were measured before and after a four-week isoenergetic MedDiet. Sex differences were found in response to the MedDiet for the proportion of medium LDL (255–260 Å) (*p* for sex-by-time interaction = 0.01) and small, dense LDL (sdLDL; <255 Å) (trend; *p* for sex-by-time interaction = 0.06), men experiencing an increase in the proportion of medium LDL with a concomitant reduction in the proportion of sdLDL, while an opposite trend was observed in women. A sex difference was also noted for estimated cholesterol concentrations among sdLDL (*p* for sex-by-time interaction = 0.03), with only men experiencing a reduction in response to the MedDiet. The MedDiet marginally reduced oxidized LDL (oxLDL) concentrations (*p* = 0.07), with no sex difference. Results suggest that short-termconsumption of the MedDiet leads to a favorable redistribution of LDL subclasses from smaller to larger LDL only in men. These results highlight the importance of considering sex issues in cardiovascular benefits of the MedDiet.

## 1. Introduction

Lowering LDL-C concentration is the primary target of therapy for the prevention of cardiovascular disease (CVD) [1,2,3]. However, in addition to LDL-C concentrations, it has been shown that a more detailed analysis of LDL physico-chemical properties (e.g., size and oxidation) provides further insight into individual cardiovascular risk [4,5,6]. Individuals characterized by a predominance of small, dense LDL particles (sdLDL) are at increased risk of coronary heart disease compared to those with larger, buoyant LDL particles [7,8,9]. Compared with large LDL, sdLDL possess a lower affinity for the LDL receptor and a longer half-life in plasma [10], bind more tightly to arterial proteoglycans [11], penetrate the arterial subendothelial space more easily [12] and are more susceptible to oxidation [13]. LDL oxidation is another process through which LDL contribute to atherosclerotic plaque formation by favoring endothelial dysfunction, the release of inflammatory cytokines and macrophage transformation into foam cells [6]. Accordingly, oxidized LDL (oxLDL) concentrations have been identified as an important marker of atherosclerotic lesions [5,6]. 

Sex disparities have been previously reported for LDL physico-chemical properties, men being characterized by a higher proportion of sdLDL and greater concentrations of oxLDL than premenopausal women [14,15,16,17,18,19]. Given that the presence of both sdLDL and oxLDL are predictive of an increased cardiovascular risk, such sex differences could contribute in part to the higher risk of coronary heart disease found in men compared with premenopausal women. 

Adopting the traditional Mediterranean diet (MedDiet) has been identified as a useful strategy in the prevention of cardiovascular events. The Prevención con Dieta Mediterránea (PREDIMED) study, consisting of a nutritional intervention among 7447 high-risk individuals, indicates that the adherence to an energy-unrestricted MedDiet supplemented with extra-virgin olive oil or mixed nuts for 4.8 years reduces by approximately 30% the incidence of myocardial infarction, stroke and cardiovascular death compared with a low-fat diet [20]. Different mechanisms of action have been proposed for the beneficial cardioprotective effects of the MedDiet, one largely documented being its LDL-C lowering effects [21]. Nevertheless, strong evidence suggests beneficial effects of the MedDiet beyond its impact on LDL-C and other traditional risk factors [22]. 

A shift toward larger LDL particles and a reduction in oxLDL concentrations have been previously observed with the consumption of the MedDiet in some [23,24,25,26,27], but not all studies [28], suggesting that some factors, such as the characteristics of participants (e.g., sex, age, genetic predisposition, usual dietary intakes) may modulate these effects. In this sense, previous results suggest that men have greater cardiometabolic changes in response to the MedDiet than premenopausal women [29,30,31]. However, no study has examined whether sex differences exist in the effects of this food pattern on LDL physico-chemical properties. One could speculate that, because of the higher proportion of sdLDL habitually found in men compared with premenopausal women [14,15,16,17], men may experience a more important shift toward larger LDL particles than women in response to the MedDiet. Consequently, since larger, less dense LDL particles are less susceptible to oxidation than sdLDL [13], men may have greater reduction in oxLDL concentrations than women. As estrogens exert beneficial effects on LDL size and oxidation [18,32,33,34,35] and the MedDiet has been previously shown to reduce estrogen concentrations in women [36], these facts further support the hypothesis that men may benefit more than premenopausal women from this food pattern.

The aim of the present study was to verify whether the impact of the MedDiet on LDL size distribution, as well as on oxLDL concentrations differs between men and women. Results presented in this paper suggest that adhering to the MedDiet, in addition to a clinically-relevant reduction in LDL-C concentrations, also has additional positive effects on LDL particle size phenotype, leading to a favorable redistribution from smaller to larger LDL in men, but not in women. This study provides new and useful clinical information in order to improve our understanding of the variability in the response to the MedDiet and, ultimately, to further individualize dietary recommendations in the prevention of cardiovascular disease.

## 2. Experimental Section 

### 2.1. Subjects

Thirty-eight men and 32 premenopausal women took part to this study. However, one man was excluded from analyses, because of a lack of dietary compliance due to illness during several days just before the end of the controlled MedDiet phase. Analyses were therefore conducted in a sample of 37 men and 32 premenopausal women, aged between 24 and 53 years, who participated in a study that was initially designed to directly document sex differences in the impact of a MedDiet on LDL-C concentrations [29]. The main inclusion criteria were to have slightly elevated LDL-C concentrations (between 3.4 and 4.9 mmol/L) or a total cholesterol to HDL-C ratio ≥5.0, as well as at least one of the four following CVD risk factors: waist circumference >94 cm in men and >80 cm in women [37]; TG ≥1.7 mmol/L; fasting glycemia between 6.1 and 6.9 mmol/L and/or blood pressure levels ≥130/85 mm Hg. No participant had a history of cardiovascular events, and none were taking medication that could affect the dependent variables under study (e.g., lipid-lowering medication). Smokers and pregnant women or those using systemic hormonal contraceptives were excluded from this study. The present study was conducted according to the guidelines laid down in the Declaration of Helsinki, and all procedures involving human subjects were approved by the Laval University Ethics Committee (#2007-180, 4 October 2007). This clinical trial was registered at www.clinicaltrials.gov as NCT01293344.

Before the intervention (*i.e.*, at screening), no difference between men and women was found for age (mean (SD): men 42.5 (7.3) years; women 41.1 (7.4) years, *p* for sex difference = 0.45) and BMI (mean (SD): men 28.8 (3.1) kg/m^2^; women 29.2 (5.6) kg/m^2^, *p* for sex difference = 0.92). However, men had higher mean values than women for body weight (mean (SD): men 90.7 (13.7) kg; women 77.4 (14.7) kg, *p* for sex difference <0.001) and waist circumference (mean (SD): men 102.1 (9.9) cm; women 94.3 (10.3) cm, *p* for sex difference = 0.001). 

Power analyses for repeated measures and within-between interactions showed that a total sample size of *n* = 69 is sufficient to detect significant differences in all outcomes measured with a small effect size estimate (Cohen’s d of 0.20 [38]) and with an α = 0.05 and a power of 0.8 (G*Power Version 3.0.10, Franz Faul, Universität Kiel, Germany).

### 2.2. Study Design

The study protocol consisted of a 4-week run-in period, immediately followed by a 4-week fully-controlled MedDiet phase [29]. During the run-in period, subjects received personalized recommendations by a registered dietitian in order to follow the healthy recommendations of Canada’s Food Guide [39]. Briefly, Canada’s Food Guide is an educational tool that promotes healthy eating for Canadians in order to reduce the risk of chronic diseases and to achieve overall health. It indicates the recommended number of food guide servings per day for each of the four food groups (vegetables and fruits, grain products, milk and alternatives and meat and alternatives) according to the age and sex of individuals. In addition, more specific recommendations are provided for each food groups (e.g., eat at least one dark green and one orange vegetable each day, make at least half of your grain products whole grain each day, select lower fat milk and alternatives, have meat alternatives, such as beans, lentils and tofu, often and eat at least two servings of fish each week). The 4-week run-in period allowed comparing the effects of the MedDiet between men and women having similar baseline dietary intakes, as reported previously [29]. Moreover, the concordance of men’s and women’s diet with the traditional MedDiet was similar at the end of the run-in period, as suggested by a Mediterranean score (MedScore; from 0–44 points: 24.8 (5.9) points in men and 24.6 (4.4) points in women, *p* for sex difference = 0.87) [40]. This MedScore has been previously shown to be a valid indicator of MedDiet adherence [40,41]. A MedScore of forty-four would imply a food pattern that is perfectly concordant with the traditional MedDiet. No change in body weight was found during the run-in period (+0.02 (0.19) kg in men and −0.01 (0.16) kg in women, respectively *p* = 0.93 and *p* = 0.84).

During the 4-week fully-controlled feeding phase, subjects consumed an experimental MedDiet formulated to be concordant with the characteristics of the traditional MedDiet [42]. More precisely, the experimental MedDiet included an abundance of fruits, vegetables, whole grain cereals, nuts and legumes, moderate amounts of fish, poultry, eggs and low-fat dairy products and low amounts of red meat and sweets. Olive oil was the main source of fat, and wine accompanied meals with moderation. The percentages of energy derived from lipids, carbohydrates, proteins and alcohol were respectively 32% (7.2% SFA, 17.9% MUFA and 4.6% PUFA), 46%, 17% and 5%. Details about the composition of the MedDiet have been already reported [29].

A 4-week controlled phase has been demonstrated as sufficient to induce significant changes in LDL particle size features [43,44] and oxLDL concentrations [45,46]. All foods for the MedDiet phase were prepared in a standardized manner in the Clinical Investigation Unit (CIU). Subjects were instructed to consume only the foods and beverages provided to them, which corresponded to 100% of their estimated energy needs. Energy needs were established by averaging energy requirements estimated by a validated food frequency questionnaire (FFQ) [47] administrated at the beginning of the run-in period and energy needs as determined by the Harris–Benedict formula. Participant’s body weight was monitored daily before the consumption of the lunch at the CIU, and energy intake was increased or decreased by 250 kcal/day if a subject lost or gained greater than 1 kg and maintained that body weight for at least 3 days. Participants completed a daily checklist confirming the consumption of provided foods and beverages and, if needed, the amount of foods not consumed. Participants were instructed to maintain their usual physical activity level. However, in order to verify whether participants followed this instruction, daily energy expenditure from physical activity participation was evaluated using a validated 3-day activity diary record developed by Bouchard *et al.* [48] administrated during the fourth week of both the run-in period and the MedDiet phase. Since some studies have suggested that fluctuations in female hormones influence the lipid-lipoprotein profile [49], women’s feeding was shortened or prolonged if needed in order to be able to carry out all tests in the early follicular phase of their menstrual cycle (from the third to the ninth day of the menstrual cycle; mean duration of the feeding period in women (SD): 28.8 (4.3) days).

### 2.3. Laboratory Analyses

Blood samples were collected from an antecubital vein into vacutainer tubes after a 12-h overnight fast. Total plasma cholesterol, TG and HDL-C concentrations were measured using commercial reagents on a Modular P chemistry analyzer (Roche Diagnostics, Mannheim, Germany). Apo B was measured by immunoturbidimetry (Roche Diagnostics, Mannheim, Germany). LDL-C was obtained by the equation of Friedewald [50]. Plasma apo A-1 and apo A-2 concentrations were measured by immunonephelometry. For the measurements of LDL particle size features and oxLDL concentrations, analyses were performed using plasma stored at −80 °C. Non-denaturing 2%–16% polyacrylamide gradient gel electrophoreses were used to characterize LDL particle size distribution, as previously described [51]. LDL particle size was computed on the basis of the relative migration of plasma standards of known diameter [52]. The LDL peak particle size was computed as the estimated diameter for the major peak of each scan. An integrated (or mean) LDL size, corresponding to the weighed mean size of all LDL subclasses in each individual, was also determined. As revealed by the analysis of pooled plasma standards, measurements of LDL peak particle size and LDL integrated particle size were highly reproducible, considering an inter-assay coefficient of variation of <2%. The relative proportion of sdLDL, characterized by a diameter <255 Å (LDL_<255Å_), was obtained by computing the relative area of the densitometric scan <255 Å. The absolute concentration of cholesterol in sdLDL particles was estimated by multiplying the total plasma LDL-C concentrations by the relative proportion of LDL_<255Å_. Similar approaches were used to estimate the relative proportion of medium and large LDL particles and their specific estimated cholesterol concentration, using respectively a diameter between 255 and 260 Å (LDL_255–260Å_) and >260 Å (LDL_>260Å_). LDL subclass Pattern A was characterized by an LDL peak particle size ≥255 Å, whereas LDL subclass Pattern B by a LDL peak particle size <255 Å. The measurements of the proportion of medium LDL and large LDL had a coefficient of variation of 12% and 9.3% respectively. oxLDL concentrations were measured using a commercial enzyme-linked immune-sorbent assay (ELISA) kit (Alpco, Salem, NH), with an intra-assay coefficient of variation of 3.9%–5.7% and inter-assay coefficient of variation of 9%–11%.

### 2.4. Statistical Analyses

Statistical analyses were performed using SAS statistical package Version 9.4 (SAS Institute Inc., Cary, NC, USA). All statistical tests were two-sided. *p* ≤ 0.05 was considered as significant. Data were collected before (*i.e.*, immediately after the run-in period, referred as baseline values) and after the controlled MedDiet phase. Differences in baseline characteristics between men and women were assessed using Student’s *t*-test. Mixed procedures for repeated measurements were used to assess the main effects of time, sex and sex-by-time interaction on LDL particle size features and oxLDL concentrations. When a significant main effect was found, Tukey-Kramer adjusted *p*-values were used to identify the precise location of differences. Associations between variables were assessed by Pearson’s correlation analyses. One woman was excluded from analyses of oxLDL concentrations due to a baseline extreme value (*i.e.*, 3027.8 ng/mL *vs.* group’s mean (SD) of 166.4 (200.6) ng/mL).

Although the MedDiet phase aimed at being isoenergetic, both men and women experienced a small, but significant body weight change (mean (SD): −1.19 (1.23) kg in men, *p* < 0.001 and −0.55 (0.98) kg in women, *p* = 0.01). Given that changes in body weight may influence LDL physico-chemical properties (both size and oxidation) [5,53], all analyses presented here were adjusted for this small change in body weight. Waist circumference did not statistically change during the MedDiet phase in both men and women (mean (SD): −0.29 (2.68) cm in men, *p* = 0.56; −0.80 (2.56) cm in women, *p* = 0.09).

## 3. Results 

Even if there were no difference between men and women for LDL-C concentrations before the MedDiet (*i.e.*, immediately after the run-in period; *p* = 0.36), some sex differences were observed in LDL physico-chemical properties (Table 1). In fact, men had a lower proportion of medium LDL_(__255–260__Å)_ (*p* = 0.02) and a higher proportion of sdLDL_(<__255__Å)_ (*p* = 0.01) than women. Moreover, men had higher estimated cholesterol concentrations among sdLDL_(<__255__Å)_ than women (*p* = 0.01). Finally, men were characterized by smaller LDL peak particle size (*p* = 0.04) and LDL integrated size (*p* = 0.04) than women. No difference was found between men and women for all of the other variables related to LDL particle size features (*p* > 0.08). Men and women had similar oxLDL concentrations prior to the controlled MedDiet intervention (*p* = 0.86).

### 3.1. Plasma Lipids and Lipoproteins

As reported previously [29], reductions in total cholesterol, LDL-C, HDL-C, total cholesterol to HDL-C ratio and apo B were observed in response to the MedDiet, and no difference was found between men and women (*p* for sex-by-time interaction ≥0.16; Table 1). More precisely, reductions in LDL-C concentrations of 10.4% in men and 7.3% in women were noted (respectively, *p* = 0.003 and *p* = 0.04). 

**Table 1:** Effects of the four-week Mediterranean diet (MedDiet) on the lipid-lipoprotein profile and LDL physical properties in men and women.

Variables	Men (*n* = 37)					Women (*n* = 32)					*p*-value ^a^	
	Baseline ^b^		After MedDiet		Change	Baseline ^b^		After MedDiet		Change	Time	Sex * time
	Mean	SEM	Mean	SEM	%	Mean	SEM	Mean	SEM	%		
TG (mmol/L) ^c,d^	1.86	0.19	1.59	0.10	−14.6	1.36	0.11	1.26	0.08	−7.7	0.06	0.53
Total cholesterol (mmol/L) ^c^	5.56	0.15	5.01	0.13	−9.9	5.40	0.11	5.06	0.10	−6.2	<0.001	0.16
LDL-C (mmol/L) ^c^	3.61	0.12	3.23	0.11	−10.4	3.47	0.09	3.22	0.09	−7.3	<0.001	0.34
HDL-C (mmol/L) ^c,d^	1.09	0.05	1.05	0.04	−4.4	1.30	0.05	1.27	0.04	−2.6	0.02	0.57
Total cholesterol/HDL-C ratio ^c^	5.30	0.17	4.97	0.17	−6.1	4.26	0.14	4.08	0.12	−4.3	0.001	0.36
Apo B (g/L) ^c^	1.14	0.04	1.03	0.04	−9.5	1.04	0.03	0.95	0.03	−9.0	<0.001	0.69
LDL physical properties												
LDL peak particle diameter (Å) ^d^	253.2	0.5	253.3	0.4	0.0	254.7	0.6	254.4	0.5	−0.1	0.79	0.42
LDL integrated size (Å) ^d^	254.2	0.4	254.4	0.3	0.1	255.5	0.5	255.4	0.4	−0.1	0.70	0.25
Relative distribution among LDL subclasses												
Large LDL_>260Å_ (%) ^d^	17.0	2.0	16.5	1.6	−0.5	22.3	2.7	20.8	2.8	−1.5	0.41	0.57
Medium LDL_255–260Å_ (%)	26.9	1.5	29.9	1.4	3.0	32.7	2.1	31.5	1.7	−1.2	0.25	0.01
Small LDL_<255Å_ (%)	56.2	2.9	53.6	2.6	−2.5	45.0	3.4	47.7	3.0	2.6	0.97	0.06
Absolute concentration of cholesterol in LDL subclasses												
Large LDL-C_>260Å_ (mmol/L) ^d^	0.64	0.08	0.56	0.06	−12.6	0.77	0.09	0.71	0.11	−8.9	0.03	0.54
Medium LDL-C_255–260Å_ (mmol/L)	0.99	0.07	0.97	0.06	−1.2	1.14	0.08	1.01	0.06	−11.5	0.06	0.12
Small LDL-C_<255Å_ (mmol/L)	1.99	0.10	1.70	0.09	−14.3	1.55	0.12	1.49	0.09	−3.5	0.001	0.03
oxLDL (ng/mL) ^d^	167.2	28.7	160.6	25.8	−4.0	165.5	41.5	152.6	32.5	−7.8	0.07	0.85

TG, triglycerides; LDL-C, low-density lipoprotein-cholesterol; HDL-C, high-density lipoprotein-cholesterol; Apo, apolipoprotein; oxLDL, oxidized LDL. ^a^ All analyses are adjusted for the body weight change during the MedDiet phase; ^b^ baseline values represent those collected after the run-in period, immediately before the 4-week MedDiet; ^c^ these data have been previously reported [30]; ^d^ since these variables were not normally distributed, a transformation was performed in order to obtain a normal distribution. TG, HDL-C and oxLDL were log-transformed; LDL peak particle diameter, LDL integrated size and small LDL-C_<255__Å_ were inversed transformed; and large LDL_>260__Å_ was square transformed.

### 3.2. LDL Size Distribution

Sex differences were observed in changes in LDL size distribution in response to the MedDiet and more precisely for the proportion of medium LDL_(255–260__Å)_ (*p* for sex-by-time interaction = 0.01) and sdLDL_(<255__Å)_ (trend; *p* for sex-by-time interaction = 0.06) (Table 1). Specifically, men experienced an increase in the proportion of medium LDL with a concomitant non-significant decrease in the proportion of sdLDL (respectively, *p* = 0.03 and *p* = 0.50), while an opposite non-significant trend was observed in women (respectively, *p* = 0.74 and *p* = 0.54). No change was observed for the proportion of large LDL_(>260__Å)_ in both men and women (Table 1). These results suggest that short-term consumption of the MedDiet leads to a favorable redistribution of LDL subclasses from smaller to larger LDL only in men.

A similar LDL size distribution was found in men and in women after the MedDiet (*p* for sex difference for each proportion of LDL subclass ≥0.14), suggesting that sex differences at baseline were no longer present after the short-term consumption of the MedDiet.

### 3.3. Estimated Cholesterol Concentration among Each LDL Subclass

A sex difference was noted for cholesterol concentrations among sdLDL_(<255Å)_ (*p* for sex-by-time interaction = 0.03), with only men experiencing a reduction in response to the MedDiet (*p* < 0.001 in men and *p* = 0.88 in women; Table 1). In men, the reduction in cholesterol concentration among sdLDL correlated with concomitant reductions in TG (*r* = 0.38, *p* = 0.02), LDL-C (*r* = 0.41, *p* = 0.01) and apo B (*r* = 0.57, *p* < 0.001). The sex difference in cholesterol concentrations among sdLDL observed at baseline was no longer present after the short-term consumption of the MedDiet (*p* for sex difference ≥0.11).

Significant reductions in cholesterol concentration among large LDL_(>260Å)_ and a tendency for a decrease in cholesterol concentration among medium LDL_(__255–260Å)_ were found, and no sex differences were observed for these variables (Table 1). 

### 3.4. LDL Peak and Integrated (Mean) Size

Consumption of the MedDiet did not affect LDL peak particle size or the LDL integrated size in both men and women (Table 1). 

**Figure 1:**
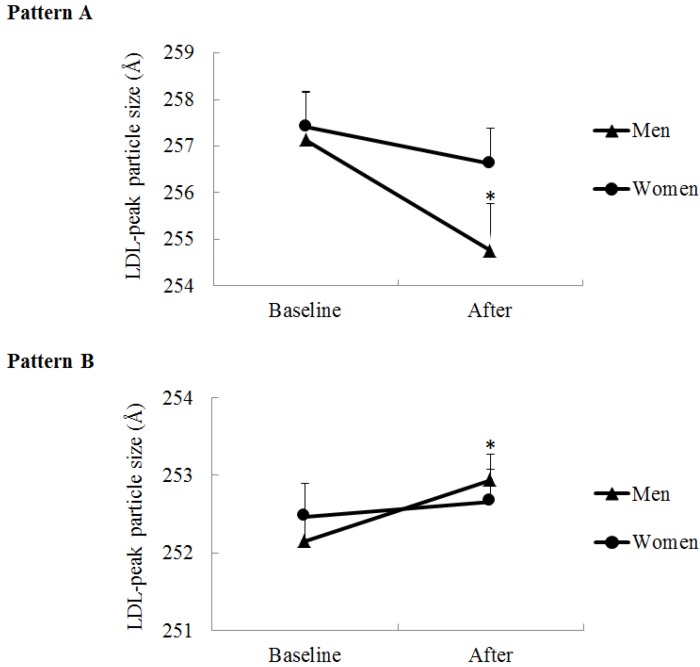
LDL peak particle size at baseline (*i.e.*, immediately before the MedDiet) and after the four-week MedDiet in men and women according to their initial LDL subclass pattern (Pattern A: LDL peak particle diameter ≥255 Å, men *n* = 8 and women *n* = 14, *p* for sex-by-time interaction = 0.12; Pattern B: LDL peak particle diameter <255 Å, men *n* = 29 and women *n* = 18, *p* for sex-by-time interaction = 0.08). Data are means (SEM). * Different from baseline in men, *p* < 0.05 by mixed procedure followed by the Tukey-Kramer test.

However, a three-way sex-by-time-by-LDL subclass pattern (A or B) interaction was found for LDL peak particle size (*p* for interaction = 0.01). Subgroup analysis indicated that, among men and women with LDL Pattern A at baseline (LDL peak particle size ≥255 Å), only men experienced a decrease in LDL peak particle size in response to the MedDiet (*p* = 0.03 for men and *p* = 0.54 for women; *p* for time effect = 0.004; *p* for sex-by-time interaction = 0.12) (Figure 1). Moreover, among those with LDL Pattern B at baseline (LDL peak particle size <255 Å), LDL peak particle size was increased in men only (*p* = 0.003 for men and *p* = 0.90 for women; *p* for time effect = 0.007; *p* for sex-by-time interaction = 0.08). Among each subgroup, no difference between men and women was found for baseline LDL peak particle size (*p* for sex difference at baseline > 0.99 for Pattern A subgroup and 0.94 for Pattern B subgroup). These results suggest that LDL subclass pattern at baseline influences the LDL peak particle size response to the MedDiet, but only in men. No three-way interaction was found for the LDL integrated size. 

Since TG concentrations are the main determinant of LDL size [4] and TG reduction in men was twice the reduction in women in response to the MedDiet (respectively −14.6% and −7.7%; Table 1), we thereafter adjusted the analyses for changes in TG concentrations. Sex differences in changes in LDL particle size features in response to the MedDiet were still observed after these adjustments (not shown). 

Adjustments for waist circumference at baseline did not influence the results obtained (not shown), suggesting that differences between men and women in abdominal obesity do not explain sex differences observed in LDL size response to the MedDiet.

### 3.5. Oxidized LDL

A trend for a reduction in oxLDL concentrations was found in response to the MedDiet, and no difference was observed between men and women (*p* for time effect = 0.07; *p* for sex-by-time interaction = 0.85; Table 1).

Diet-induced changes in oxLDL concentrations were not associated with oxLDL concentrations at baseline in either men or women (respectively *r* = −0.06, *p* = 0.71 and *r* = −0.16, *p* = 0.40). There was no association between changes in oxLDL concentrations and changes in LDL-C concentrations (respectively *r* = 0.20, *p* = 0.25 for men and *r* = 0.13, *p* = 0.50 for women) and changes in cholesterol concentration among sdLDL_(<255__Å)_ (*r* = 0.11, *p* = 0.54 for men and *r* = 0.08, *p* = 0.67 for women). 

Although participants were instructed to maintain their usual physical activity level, a similar decrease in daily energy expenditure from physical activity participation was observed during the MedDiet phase in men and women (−27.8% in men and −27.6% in women, *p* for sex-by-time interaction = 0.25). Adjustment for changes in physical activity participation did not influence the results obtained (not shown).

## 4. Discussion 

Elevated LDL-C concentration has been established as a major risk factor for CVD [1,2,3]. However, a more detailed analysis of LDL physico-chemical properties is highly relevant given the important atherogenicity of sdLDL particles and of oxLDL [4,5,6]. Accordingly, results from the present study highlight sex-specific cardiovascular benefits of the MedDiet, which would not have been observed if only LDL-C concentrations had been measured. Indeed, despite the fact that men and women have similar reductions in LDL-C concentrations in response to the MedDiet, different responses in LDL particle size features to this food pattern are observed. More precisely, men experienced an increase in the proportion of medium LDL particles with a concomitant reduction in the proportion of sdLDL, while an opposite non-significant trend was observed in women. Moreover, a reduction in cholesterol concentrations among sdLDL with MedDiet was noted in men, but not in women. Finally, results showed that the more deteriorated LDL particle size features found in men compared with premenopausal women at baseline were no longer present after the short-term consumption of the MedDiet. 

Even if a favorable LDL size redistribution and a reduction in cholesterol concentration among sdLDL were observed in men, one might question the clinical relevance of these changes, since the MedDiet had no global impact on LDL peak particle size, a widely-used surrogate marker of sdLDL. However, a prospective study including 2,034 middle-aged men suggested that LDL peak particle size is a weak predictor of ischemic heart disease risk compared with the proportion and the cholesterol content of sdLDL, which were identified as very powerful and independent risk predictors, even after adjustments for non-lipid and other lipid CVD risk factors, including LDL-C, HDL-C, TG and lipoprotein(a) (Lp(a)) concentrations [51]. Therefore, one mechanism behind the reduction in cardiovascular events with the consumption of the MedDiet could be a reduction of sdLDL in dyslipidemic men.

As previously found in the literature, our results highlight that, compared with premenopausal women, age-matched men have a greater proportion of sdLDL [14,15,16,17]. Given their greater proportion of sdLDL at baseline, men had more room for improvement, which may explain at least partly why they benefited more from the MedDiet than premenopausal women. Indeed, in previous studies, those including subjects with metabolic syndrome, which is a cluster of metabolic abnormalities characterized by a predominance of sdLDL, found increases in LDL size and/or a favorable redistribution of cholesterol among each LDL subclass with the MedDiet [23,24,25], while one studying the impact of the MedDiet in healthy individuals has observed no effect [28]. In line with these previous studies, strong negative correlations were found in the present study between the proportion and the cholesterol content of sdLDL at baseline and changes in these specific variables in response to the MedDiet, meaning that individuals who were characterized by a higher proportion and cholesterol content of sdLDL benefited more from the MedDiet (respectively, *r* < −0.50 and *r* < −0.61, *p* < 0.001). Additional analyses also showed that, when men and women were individually matched for the proportion of sdLDL at baseline (23 men and 23 women), differences between men and women in changes in cholesterol concentration among sdLDL were considerably smaller than the one found with the whole sample (−10.4% in men and −7.9% in women, *p* for sex-by-time interaction = 0.68 in subjects paired for the proportion of sdLDL at baseline; *vs.* −14.3% in men and −3.5% in women when the whole sample was considered). 

In addition to the difference between men and women in LDL particle size features at baseline, other factors related to sex seem to influence the impact of the MedDiet on LDL size. In fact, in the present study, subjects characterized by the LDL Pattern B phenotype (*i.e.*, predominance of sdLDL) experienced a favorable increase in LDL peak particle size, whereas a reduction was found in those characterized by the LDL Pattern A phenotype (*i.e.*, predominance of large LDL particles). However, this pattern of changes was observed only in men, while women experienced no change, regardless of their baseline LDL peak particle size phenotype. Since these sub-analyses are based on small numbers of subjects in each group, they need to be confirmed. 

Although not significant from a statistical perspective, men experienced nearly a two-fold greater reduction in TG concentrations compared with women (14.6% *vs.* 7.7%), and such a difference could be clinically significant. High concentrations of TG favor the production of large, TG-rich, very low density lipoprotein (VLDL), a precursor of TG-rich LDL. In turn, lipolysis of TG in TG-enriched LDL by hepatic lipase leads to the formation of sdLDL [4]. One could therefore assume that the greater reduction in the concentrations of sdLDL found in men compared with women in response to the MedDiet could also be explained by this greater reduction in TG concentrations in men. In the present study, a strong association between changes in sdLDL and concomitant changes in TG concentrations was found in men. However, sex differences in LDL particle size feature responses to the MedDiet were still observed after adjustments for changes in TG concentrations, suggesting that sex differences observed in the present study were not due only to a greater reduction in TG concentrations in men compared with women. 

The reduction in oxLDL concentrations was not associated with decreases in LDL-C concentrations in response to the MedDiet in both men and women, suggesting that oxLDL reduction does not simply reflect the LDL-lowering effects of the MedDiet, but is likely to be due to the antioxidant properties of this food pattern. Since men reduced their proportion and cholesterol content of sdLDL more than women in response to the MedDiet and sdLDL is more susceptible to oxidation compared with larger particles [13], it could be expected that men may have reduced their oxLDL concentrations to a greater extent compared with women. However, this was not the case. In fact, no sex difference was found in response to the MedDiet, with a modest reduction in oxLDL concentrations observed in both men and women. One possible explanation is that the consumption of a diet rich in oleic acid, the more abundant fatty acid in the olive oil, may reduce the susceptibility of sdLDL to oxidation [54], leading to a reduction in oxLDL, which is independent of the one found in sdLDL. This disassociation between changes in sdLDL and changes in oxLDL concentrations was observed in both men and women. Moreover, the MedDiet is rich in antioxidants, such as carotenoids, tocopherols and phenolic compounds, mainly contained in vegetables, fruits, olive oil and red wine [55], which may provide an important protection for sdLDL to oxidation. Therefore, these results suggest that the high oleic acid content of the MedDiet, along with its important antioxidant properties, may overshadow the link between sdLDL and LDL oxidation. 

One of the strengths of our study is a design that included a highly-controlled dietary phase, which permitted precisely investigating sex differences in response to the MedDiet with a maximum control of possible confounding variables. Moreover, this study included a large number of subjects for a strictly controlled feeding study. However, results should not be generalized, and additional studies are needed. The duration of the controlled phase was appropriate, considering that previous studies of equal or shorter lengths showed significant effects of diet on LDL particle size [43,44] and oxLDL concentrations [45,46]. Among the limitations, the study’s ‘single strand before and after’ design does not allow comparisons to a control diet, and therefore, non-specific treatment effects that are not attributable to the MedDiet cannot be ruled out. However, we consider that the absence of a control diet is not a major limitation, since the main objective of this study was to directly compare men’s and women’s response to the MedDiet.

## 5. Conclusions 

In conclusion, data from this strictly-controlled feeding study indicated for the first time the existence of sex differences in the response of the LDL particle size features to the MedDiet in dyslipidemic men and premenopausal women. Our results suggest that adhering to the MedDiet, in addition to a clinically-relevant reduction in LDL-C concentrations (−10.4% in men and −7.3% in women), also has additional positive effects on LDL particle size phenotype, leading to a favorable redistribution from smaller to larger LDL in men, but not in women. Moreover, men, but not women, with smaller LDL peak particle size at baseline (<255 Å) increase LDL peak particle size in response to the MedDiet. Since sdLDL has been suggested as a strong predictor of the progression of atherosclerosis and CVD events [7,8,9], such findings have implications, both for improved understanding of sex-specific mechanisms behind the beneficial cardiovascular effects of the MedDiet and for the clinical management of dyslipidemic men.

## References

[1] Anderson T.J., Gregoire J., Hegele R.A., Couture P., Mancini G.B., McPherson R., Francis G.A., Poirier P., Lau D.C., Grover S. (2013). 2012 update of the canadian cardiovascular society guidelines for the diagnosis and treatment of dyslipidemia for the prevention of cardiovascular disease in the adult. Can. J. Cardiol..

[2] Reiner Z., Catapano A.L., de Backer G., Graham I., Taskinen M.R., Wiklund O., Agewall S., Alegria E., European Association for Cardiovascular Prevention and Rehabilitation (2011). ESC/EAS guidelines for the management of dyslipidaemias: The task force for the management of dyslipidaemias of the european society of cardiology (ESC) and the european atherosclerosis society (EAS). Eur. Heart J..

[3] National Cholesterol Education Program (NCEP) Expert Panel on Detection, Evaluation, and Treatment of High Blood Cholesterol in Adults (Adult Treatment Panel III) (2002). Third report of the national cholesterol education program (NCEP) expert panel on detection, evaluation, and treatment of high blood cholesterol in adults (Adult Treatment Panel III) final report. Circulation.

[4] Mikhailidis D.P., Elisaf M., Rizzo M., Berneis K., Griffin B., Zambon A., Athyros V., de Graaf J., Marz W., Parhofer K.G. (2011). “European panel on low density lipoprotein (LDL) subclasses”: A statement on the pathophysiology, atherogenicity and clinical significance of LDL subclasses. Curr. Vasc. Pharmacol..

[5] Ishigaki Y., Oka Y., Katagiri H. (2009). Circulating oxidized LDL: A biomarker and a pathogenic factor. Curr. Opin. Lipidol..

[6] Mitra S., Goyal T., Mehta J.L. (2011). Oxidized ldl, lox-1 and atherosclerosis. Cardiovasc. Drugs Ther..

[7] Hoogeveen R.C., Gaubatz J.W., Sun W., Dodge R.C., Crosby J.R., Jiang J., Couper D., Virani S.S., Kathiresan S., Boerwinkle E. (2014). Small dense low-density lipoprotein-cholesterol concentrations predict risk for coronary heart disease: The atherosclerosis risk in communities (ARIC) study. Arterioscler. Thromb. Vasc. Biol..

[8] Lamarche B., St-Pierre A.C., Ruel I.L., Cantin B., Dagenais G.R., Despres J.P. (2001). A prospective, population-based study of low density lipoprotein particle size as a risk factor for ischemic heart disease in men. Can. J. Cardiol..

[9] Lamarche B., Tchernof A., Moorjani S., Cantin B., Dagenais G.R., Lupien P.J., Despres J.P. (1997). Small, dense low-density lipoprotein particles as a predictor of the risk of ischemic heart disease in men. Prospective results from the quebec cardiovascular study. Circulation.

[10] Nigon F., Lesnik P., Rouis M., Chapman M.J. (1991). Discrete subspecies of human low density lipoproteins are heterogeneous in their interaction with the cellular LDL receptor. J. Lipid Res..

[11] Camejo G., Hurt-Camejo E., Wiklund O., Bondjers G. (1998). Association of apo B lipoproteins with arterial proteoglycans: Pathological significance and molecular basis. Atherosclerosis.

[12] Bjornheden T., Babyi A., Bondjers G., Wiklund O. (1996). Accumulation of lipoprotein fractions and subfractions in the arterial wall, determined in an *in vitro* perfusion system. Atherosclerosis.

[13] De Graaf J., Hak-Lemmers H.L., Hectors M.P., Demacker P.N., Hendriks J.C., Stalenhoef A.F. (1991). Enhanced susceptibility to *in vitro* oxidation of the dense low density lipoprotein subfraction in healthy subjects. Arterioscler. Thromb..

[14] Vekic J., Zeljkovic A., Jelic-Ivanovic Z., Spasojevic-Kalimanovska V., Bogavac-Stanojevic N., Memon L., Spasic S. (2009). Small, dense LDL cholesterol and apolipoprotein B: Relationship with serum lipids and LDL size. Atherosclerosis.

[15] Lemieux I., Pascot A., Lamarche B., Prud’homme D., Nadeau A., Bergeron J., Despres J.P. (2002). Is the gender difference in LDL size explained by the metabolic complications of visceral obesity?. Eur. J. Clin. Investig..

[16] Li Z., McNamara J.R., Fruchart J.C., Luc G., Bard J.M., Ordovas J.M., Wilson P.W., Schaefer E.J. (1996). Effects of gender and menopausal status on plasma lipoprotein subspecies and particle sizes. J. Lipid Res..

[17] Nikkila M., Pitkajarvi T., Koivula T., Solakivi T., Lehtimaki T., Laippala P., Jokela H., Lehtomaki E., Seppa K., Sillanaukee P. (1996). Women have a larger and less atherogenic low density lipoprotein particle size than men. Atherosclerosis.

[18] Miller A.A., de Silva T.M., Jackman K.A., Sobey C.G. (2007). Effect of gender and sex hormones on vascular oxidative stress. Clin. Exp. Pharmacol. Physiol..

[19] Wu T., Willett W.C., Rifai N., Shai I., Manson J.E., Rimm E.B. (2006). Is plasma oxidized low-density lipoprotein, measured with the widely used antibody 4E6, an independent predictor of coronary heart disease among U.S. Men and women?. J. Am. Coll. Cardiol..

[20] Estruch R., Ros E., Martinez-Gonzalez M.A. (2013). Mediterranean diet for primary prevention of cardiovascular disease. N. Eng. J. Med..

[21] Serra-Majem L., Roman B., Estruch R. (2006). Scientific evidence of interventions using the mediterranean diet: A systematic review. Nutr. Rev..

[22] Delgado-Lista J., Perez-Martinez P., Garcia-Rios A., Perez-Caballero A.I., Perez-Jimenez F., Lopez-Miranda J. (2014). Mediterranean diet and cardiovascular risk: Beyond traditional risk factors. Crit. Rev. Food Sci. Nutr..

[23] Richard C., Couture P., Ooi E.M., Tremblay A.J., Desroches S., Charest A., Lichtenstein A.H., Lamarche B. (2014). Effect of mediterranean diet with and without weight loss on apolipoprotein B100 metabolism in men with metabolic syndrome. Arterioscler. Thromb. Vasc. Biol..

[24] Damasceno N.R., Sala-Vila A., Cofan M., Perez-Heras A.M., Fito M., Ruiz-Gutierrez V., Martinez-Gonzalez M.A., Corella D., Aros F., Estruch R. (2013). Mediterranean diet supplemented with nuts reduces waist circumference and shifts lipoprotein subfractions to a less atherogenic pattern in subjects at high cardiovascular risk. Atherosclerosis.

[25] Jones J.L., Comperatore M., Barona J., Calle M.C., Andersen C., McIntosh M., Najm W., Lerman R.H., Fernandez M.L. (2012). A mediterranean-style, low-glycemic-load diet decreases atherogenic lipoproteins and reduces lipoprotein (a) and oxidized low-density lipoprotein in women with metabolic syndrome. Metabolism.

[26] Lapointe A., Goulet J., Couillard C., Lamarche B., Lemieux S. (2005). A nutritional intervention promoting the mediterranean food pattern is associated with a decrease in circulating oxidized LDL particles in healthy women from the quebec city metropolitan area. J. Nutr..

[27] Fito M., Guxens M., Corella D., Saez G., Estruch R., de la T.R., Frances F., Cabezas C., Lopez-Sabater M.C., Marrugat J. (2007). Effect of a traditional mediterranean diet on lipoprotein oxidation: A randomized controlled trial. Arch. Intern. Med..

[28] Goulet J., Lamarche B., Charest A., Nadeau G., Lapointe A., Desroches S., Lemieux S. (2004). Effect of a nutritional intervention promoting the mediterranean food pattern on electrophoretic characteristics of low-density lipoprotein particles in healthy women from the quebec city metropolitan area. Br. J. Nutr..

[29] Bedard A., Riverin M., Dodin S., Corneau L., Lemieux S. (2012). Sex differences in the impact of the mediterranean diet on cardiovascular risk profile. Br. J. Nutr..

[30] Bedard A., Corneau L., Lamarche B., Dodin S., Lemieux S. (2014). Sex-related differences in the effects of the mediterranean diet on glucose and insulin homeostasis. J. Nutr. Metab..

[31] Bedard A., Tchernof A., Lamarche B., Corneau L., Dodin S., Lemieux S. (2014). Effects of the traditional mediterranean diet on adiponectin and leptin concentrations in men and premenopausal women: Do sex differences exist?. Eur. J. Clin. Nutr..

[32] Packard C., Caslake M., Shepherd J. (2000). The role of small, dense low density lipoprotein (LDL): A new look. Int. J. Cardiol..

[33] Schaefer E.J., Foster D.M., Zech L.A., Lindgren F.T., Brewer H.B., Levy R.I. (1983). The effects of estrogen administration on plasma lipoprotein metabolism in premenopausal females. J. Clin. Endocrinol. Metab..

[34] Law J., Bloor I., Budge H., Symonds M.E. (2014). The influence of sex steroids on adipose tissue growth and function. Horm. Mol. Biol. Clin. Investig..

[35] Arias-Loza P.A., Muehlfelder M., Pelzer T. (2013). Estrogen and estrogen receptors in cardiovascular oxidative stress. Pflug. Arch..

[36] Carruba G., Granata O.M., Pala V., Campisi I., Agostara B., Cusimano R., Ravazzolo B., Traina A. (2006). A traditional mediterranean diet decreases endogenous estrogens in healthy postmenopausal women. Nutr. Cancer.

[37] International Diabetes Federation (2010). IDF Worldwide Definition of the Metabolic Syndrome. http://www.idf.org/webdata/docs/MetS_def_update2006.pdf.

[38] Bird K.D. (2002). Confidence intervals for effect sizes in analysis of variance. Educ. Psychol. Meas..

[39] Health Canada Eating Well with Canada’s Food Guide. http://www.hc-sc.gc.ca/fn-an/food-guide-aliment/index-eng.php.

[40] Goulet J., Lamarche B., Nadeau G., Lemieux S. (2003). Effect of a nutritional intervention promoting the mediterranean food pattern on plasma lipids, lipoproteins and body weight in healthy french-canadian women. Atherosclerosis.

[41] Leblanc V., Begin C., Hudon A.M., Royer M.M., Corneau L., Dodin S., Lemieux S. (2014). Gender differences in the long-term effects of a nutritional intervention program promoting the mediterranean diet: Changes in dietary intakes, eating behaviors, anthropometric and metabolic variables. Nutr. J..

[42] Willett W.C., Sacks F., Trichopoulou A., Drescher G., Ferro-Luzzi A., Helsing E., Trichopoulos D. (1995). Mediterranean diet pyramid: A cultural model for healthy eating. Am. J. Clin. Nutr..

[43] Kratz M., Gulbahce E., von Eckardstein A., Cullen P., Cignarella A., Assmann G., Wahrburg U. (2002). Dietary mono- and polyunsaturated fatty acids similarly affect LDL size in healthy men and women. J. Nutr..

[44] Lamarche B., Desroches S., Jenkins D.J., Kendall C.W., Marchie A., Faulkner D., Vidgen E., Lapsley K.G., Trautwein E.A., Parker T.L. (2004). Combined effects of a dietary portfolio of plant sterols, vegetable protein, viscous fibre and almonds on ldl particle size. Br. J. Nutr..

[45] Kay C.D., Gebauer S.K., West S.G., Kris-Etherton P.M. (2010). Pistachios increase serum antioxidants and lower serum oxidized-LDL in hypercholesterolemic adults. J. Nutr..

[46] Avellone G., Di Garbo V., Campisi D., de Simone R., Raneli G., Scaglione R., Licata G. (2006). Effects of moderate sicilian red wine consumption on inflammatory biomarkers of atherosclerosis. Eur. J. Clin. Nutr..

[47] Goulet J., Nadeau G., Lapointe A., Lamarche B., Lemieux S. (2004). Validity and reproducibility of an interviewer-administered food frequency questionnaire for healthy french-canadian men and women. Nutr. J..

[48] Bouchard C., Tremblay A., Leblanc C., Lortie G., Savard R., Theriault G. (1983). A method to assess energy expenditure in children and adults. Am. J. Clin. Nutr..

[49] Muesing R.A., Forman M.R., Graubard B.I., Beecher G.R., Lanza E., McAdam P.A., Campbell W.S., Olson B.R. (1996). Cyclic changes in lipoprotein and apolipoprotein levels during the menstrual cycle in healthy premenopausal women on a controlled diet. J. Clin. Endocrinol. Metab..

[50] Friedewald W.T., Levy R.I., Fredrickson D.S. (1972). Estimation of the concentration of low-density lipoprotein cholesterol in plasma, without use of the preparative ultracentrifuge. Clin. Chem..

[51] St Pierre A.C., Ruel I.L., Cantin B., Dagenais G.R., Bernard P.M., Despres J.P., Lamarche B. (2001). Comparison of various electrophoretic characteristics of ldl particles and their relationship to the risk of ischemic heart disease. Circulation.

[52] Tchernof A., Lamarche B., Prud’Homme D., Nadeau A., Moorjani S., Labrie F., Lupien P.J., Despres J.P. (1996). The dense LDL phenotype. Association with plasma lipoprotein levels, visceral obesity, and hyperinsulinemia in men. Diabetes Care.

[53] Tzotzas T., Evangelou P., Kiortsis D.N. (2011). Obesity, weight loss and conditional cardiovascular risk factors. Obes Rev..

[54] Reaven P.D., Grasse B.J., Tribble D.L. (1994). Effects of linoleate-enriched and oleate-enriched diets in combination with alpha-tocopherol on the susceptibility of LDL and LDL subfractions to oxidative modification in humans. Arterioscler. Thromb..

[55] Hadziabdic M.O., Bozikov V., Pavic E., Romic Z. (2012). The antioxidative protecting role of the mediterranean diet. Coll. Antropol..

